# Fetal alcohol spectrum disorder resources for health professionals: a scoping review

**DOI:** 10.1136/bmjopen-2024-086999

**Published:** 2024-07-12

**Authors:** Thomas Stubbs, Lisa Cannon, Emily Carter, Habiba Naanai, Josephine Chidinma Okurame, Alexandra L C Martiniuk, Jadnah Davies, Sue Thomas, Mudge Bedford, Elizabeth J Elliott, Lauren J Rice

**Affiliations:** 1 Speciality of Child and Adolescent Health, University of Sydney, Faculty of Medicine and Health, Sydney, New South Wales, Australia; 2 School of Public Health, University of Sydney, Faculty of Medicine and Health, Sydney, New South Wales, Australia; 3 Marulu Unit, Marninwarntikura Women’s Resource Centre, Fitzroy Crossing, Western Australia, Australia; 4 Office of the Chief Scientist, The George Institute for Global Health, Sydney, New South Wales, Australia; 5 Dalla Lana School of Public Health, The University of Toronto, Toronto, Ontario, Canada; 6 NDIS Remote Community Connector Team, Marra Worra Worra Aboriginal Cooporation, Fitzroy Crossing, Western Australia, Australia; 7 Sydney Children's Hospital Network and Kid's Research, Westmead, Sydney, New South Wales, Australia

**Keywords:** health services, primary health care, public health

## Abstract

**Abstract:**

**Objectives:**

This scoping review aimed to identify and critically appraise resources for health professionals to identify, diagnose, refer, and support individuals with fetal alcohol spectrum disorder (FASD)—including the extent to which the resources are appropriate for use in communities with First Nations Peoples.

**Method:**

Seven peer-reviewed databases (April 2022) and 14 grey literature websites (August 2022) were searched. The reference lists of all sources that underwent full-text review were handsearched, and FASD experts were consulted for additional sources. Resources were assessed using the Appraisal of Guidelines for REsearch and Evaluation II instrument and an adapted version of the National Health and Medical Research Council FORM Framework and iCAHE Guideline Quality Checklist.

**Results:**

A total of 41 resources underwent data extraction and critical appraisal, as screening and/or diagnosis guidelines were excluded because they are covered in other reviews. Most were recently published or updated (n=24), developed in the USA (n=15, 36.6%) or Australia (n=12, 29.3%) and assisted with FASD patient referral or support (n=40). Most management guidelines scored 76%–100% on overall quality assessment (n=5/9) and were recommended for use in the Australian context with modifications (n=7/9). Most of the guides (n=15/22) and factsheets (n=7/10) received a ‘good’ overall score. Few (n=3/41) resources were explicitly designed for or with input from First Nations Australians.

**Conclusion:**

High-quality resources are available to support health professionals providing referrals and support to individuals with FASD, including language guides. Resources should be codesigned with people living with FASD to capture and integrate their knowledge and preferences.

**STRENGTHS AND LIMITATIONS OF THIS STUDY**

- First scoping review to identify and appraise publicly available resources to aid health professionals with referral and support for people with fetal alcohol spectrum disorder and to include a focus on resources for health professionals working in First Nations communities.
- The review follows the JBI Manual for Evidence Synthesis framework and uses the Preferred Reporting Items for Systematic Reviews and Meta-Analyses for Scoping Reviews checklist to improve the reporting of scoping reviews.
- The Appraisal of Guidelines for REsearch and Evaluation II instrument and a combined, modified version of the International Centre for Allied Health Evidence Guideline Quality Checklist and the National Health and Medical Research Council FORM framework, were used to critically appraise resources.
- Resources unavailable in English or requiring payment for access were excluded. Videos were gathered but did not undergo data extraction or appraisal because they did not align with the study’s data extraction and critical appraisal tools.

## Introduction

 Fetal alcohol spectrum disorder (FASD) is a diagnostic term for a condition that can result from prenatal alcohol exposure (PAE).^1^ FASD is characterised by neurodevelopmental impairment associated with a range of psychological, emotional and behavioural difficulties and congenital anomalies.^2 3^ For people with FASD, these effects can negatively impact learning, social and emotional well-being, and academic outcomes and increase the risk of mental health concerns and engagement with child protection and justice systems.^4^,^6^ However, people with FASD display strength and resilience in the face of these challenges, including self-awareness, human connection and receptivity to support.^7^ The global prevalence of FASD among children and youth in the general population is estimated to be 7.7 per 1000,^8^ with similarly high rates observed among children in Western countries like the USA,^9^ Canada^10^ and the UK.^11^ The prevalence of FASD among children in some marginalised populations is 10–40 times higher than the global estimate,^12^ suggesting that social and economic disadvantage contributes to the risk of FASD as it does with other health outcomes.

Colonisation, trauma and racism continue to negatively impact the health and well-being of First Nations Peoples, including in Australia.^13^,^16^ Although the prevalence of FASD in Australia’s general population is unknown, the Lililwan Project revealed high rates of high-risk alcohol use during pregnancy^17^ and FASD (19%) among children in remote, First Nations Australian communities in the Kimberley region of Western Australia.^18 19^ These children displayed high rates of neurodevelopmental delay,^20^,^24^ behavioural challenges^25^ and increased hospital admissions.^26^ Health services in remote communities in Australia often lack sufficient health professionals and facilities to address the increased needs of this population.^27 28^ In remote communities, young First Nations Australians with FASD may have increased contact with child protection and criminal justice systems,^29^,^32^ highlighting the importance of early diagnosis and adequate support.^33^

Health professionals are well positioned to deliver FASD prevention^34^ and facilitate integrated care, referral and support for individuals with FASD and their caregivers.^35^ However, many have limited expertise and knowledge of how to address alcohol-related harms, confidence in diagnosis and management of FASD and access to FASD-related resources, resulting in a hesitance to initiate alcohol consumption discussions with pregnant women attending their services.^36^,^40^ These challenges may be exacerbated in cross-cultural settings, where health professionals and social workers report challenges in discussing FASD and concerns for cultural appropriateness.^41^ Relatedly, there are calls for the integration of First Nations and Western wisdom into how health services engage with First Nations communities on FASD^42^ and increased First Nations leadership in the codesign of resources and campaigns for these communities.^43^

Although previous reviews have focused on the availability of FASD resources for a broad audience^44^,^49^ or education professionals,^50^ to our knowledge, no review has focused on FASD resources and standardised tools specifically tailored to health professionals.^1^ This is an important gap in the literature as research has demonstrated both the crucial role that health professional can contribute to FASD prevention^34^ and integrated care for families living with FASD^35^ and their limited capacity and confidence to do so.^36^,^40^ Further, as noted above, health professionals working in cross-cultural settings, including First Nations Australian communities,^41^ face increased challenges to engage with families around FASD. Given the high prevalence^1217^,^19^ and burden^20^,^33^ of FASD among minority and marginalised populations, equipping health professionals to play an increased and more effective role in FASD prevention and support may have major public health gains. Consequently, in this scoping review, we aimed to identify, analyse and critically appraise publicly accessible FASD resources specifically designed to assist health professionals to identify, diagnose, refer or support people with FASD. We also aimed to evaluate the appropriateness of the resources for health professionals working with First Nations communities. Our working definition of the term ‘resources’ refers to the successive itemisation of instructions in the form of frameworks, guidelines, guides, factsheets, tools, instruments, applications or models developed for FASD.

## Methods

The scoping review used a previously developed framework^51^ and reported according to the Preferred Reporting Items for Systematic Reviews and Meta-Analyses for Scoping Reviews (PRISMA-ScR),^52^ organised into nine stages based on an updated guideline for scoping reviews.^53^ Details are outlined in the scoping review protocol.^54^ An overview is described below, including changes to the published protocol.

### Stage 1: research question and objectives

The primary research question was: What resources or guidelines are available for health professionals for the diagnosis, assessment, referral (including referral for management) or management of FASD? Secondary research questions were: What is the evidence base, applicability, generalisability and overall credibility of the resource? and What is the key purpose of the resource, including screening, diagnosis, referral for treatment and other learning and psychosocial supports? After the publication of the study protocol,^54^ an additional aim was added to explore the extent to which the resources are culturally appropriate for First Nations Australians.

### Stage 2: inclusion criteria

Sources were obtained from peer-reviewed and grey literature searches and were defined as primary research studies, systematic reviews, books, policies and websites. Resources were defined as a product or output from a source, including guidelines, guides, factsheets, videos, podcasts, apps and online learning materials. The study protocol presents the inclusion criteria,^54^ including the language requirement that resources must be published in English. Consequently, non-English resources, including those written in languages for some minority or marginalised groups, were excluded from this scoping review. This limitation is addressed below.

### Stage 3: search strategy

In 2022, peer-reviewed databases (n=7) and grey literature websites (n=14) were searched to identify resources, including the Australian Department of Health (n=9) and national/international FASD organisations’ (n=5) websites. Consultations with FASD experts were then undertaken to identify other potential resources. Details are presented in tables 3 and 4 of the study protocol^54^ and online supplemental table S1. A concept table was developed for each database and website searched, consistent with the PRISMA-ScR checklist.^52^ The Medline electronic search strategy is presented in online supplemental table S2. Data were exported to EndNote reference management software and then Covidence software, where duplicates were removed.

### Stages 4–5: screening and selection

Using Covidence, screening and selection were conducted following the PRiSMA-ScR statement and checklist in three phases.^53^ Titles and abstracts were screened by one coauthor (HN) and repeat screening of 20% was conducted by another (JCO). Sources then underwent full-text screening by one coauthor (HN) to identify potentially relevant resources and repeat screening of 20% by a second (JCO). Title/abstract and full-text screening revealed high inter-rater reliability (95% and 98% agreement, respectively). Resources were also retrieved from handsearching the reference lists of sources identified during phase two of the screening. Any potentially relevant resource was imported for full-text review and retrieval of resources for data extraction.

Several published systematic reviews of FASD screening tools^45 55^ and diagnostic guidelines exist,^56^ including a registered systematic review of FASD diagnostic guidelines.^57^ Consequently, all resources that focused only on screening and/or diagnosis were excluded from data extraction and critical appraisal. Health professionals seeking information on FASD screening tools and diagnostic guidelines can access them through current and future publications.

One coauthor (TS) reviewed a selection of videos identified and deemed that their content varied greatly from that included in the study’s data extraction and critical appraisal tools so they were excluded. Consequently, data extraction was only completed for guidelines, guides and factsheets focused on referral/management or policy/broad topics.

### Stage 6: data extraction

Four coauthors (JCO, LC, HN and LJR) performed pilot data extraction from randomly selected resources to test the suitability and efficiency of the data extraction template.^58^ The template was then modified to better suit the research questions and objectives. Following the pilot, two coauthors (TS and HN) conducted data extraction using the modified version of the extraction template (table 1). Then, another coauthor (LC) reviewed 24% of the extracted resources (n=10) to check that the data extraction template had been applied appropriately and consistently, resulting in minor additions to the data extraction and a high level of agreement (96.4%).

**Table 1:** Data extraction table for FASD resources for health professionals

Category	Details
Reference	Author(s), resource title, publication/updated year and link
Type	Factsheets, guides, guidelines, videos, websites or screening tools
Format	Journal article, report or other
Purpose/aim	Overall purpose/aim of the resource for health professionals
Country of origin	Resource country of origin
Health service level	Level of health service that the resource is focused on supporting, including policy, administrative, face to face with patients
Health professional	Health professionals who are the intended audience or would benefit from using the resource
Focus	The primary objective of the resource is to support health professionals working with those with FASD with (1) screening, (2) diagnosis, (3) referral/management, (4) referral/management (specifically language guide), (5) policy/broad and (6) prevention information
Resource outcome measure(s) and/or recommendations	The resource outcome measure(s) and/or recommendations
Evidence base of the resource	Details regarding the evidence base of the resource, including none reported, expert judgement or literature/clinical research
Applicability to First Nations Australians	The resource (1) mentions how it can be used by First Nations Australians, (2) was designed specifically for health professionals working in First Nations Australian communities or (3) was designed with input from First Nations Australians

FASDfetal alcohol spectrum disorder

### Stage 7: quality appraisal

Quality appraisal of resources categorised as ‘guidelines’ was conducted using the Appraisal of Guidelines for REsearch and Evaluation II (AGREE II) instrument,^59^ with an additional ‘Applicability—First Nations Australians’ domain (four items) to align with the study’s additional focus on this population (online supplemental table S3). Weighted scores were calculated for each domain and overall scores were calculated for each resource, including a judgement as to the resource’s fit for recommendation. Other resources categorised as ‘guides’ or ‘factsheets’ were appraised using a modified appraisal,^50^ a combination of the International Centre for Allied Health Evidence (iCAHE) Guideline Quality Checklist^60^ and the National Health and Medical Research Council (NHMRC) FORM framework.^61 62^ As this scoping review had a health focus, a previously developed tool^50^ was modified to align with the NHMRC FORM framework, as shown in the appraisal tool (online supplemental table S4). A pilot was conducted in which two coauthors (JCO and LC) appraised 10 resources and discussed discrepancies with the research team. Each tool component received a grade ranging from A (excellent) to D (poor). Applicability for the Australian context was assessed to establish relevance specifically for Australian health professionals. The item on applicability to patient populations was modified to include a subcomponent on First Nations Australians, in line with the review’s additional aim. One coauthor (TS) conducted the quality appraisal of resources. Another coauthor (LC) completed the quality appraisal of 24% (n=10) of all resources that underwent data extraction, including guidelines (n=4) using the AGREE II instrument and guides (n=5) and a factsheet (n=1). The modified appraisal tool was used to ensure consistency and reliability of the process.^50^ An inter-rater reliability score of 88.9% was obtained for both quality appraisal tools. The two coauthors then met to discuss and reconcile the scoring differences.

## Results

### Stage 8-9: data reporting & evidence summary

#### Screening and selection of resources

**Figure 1:**
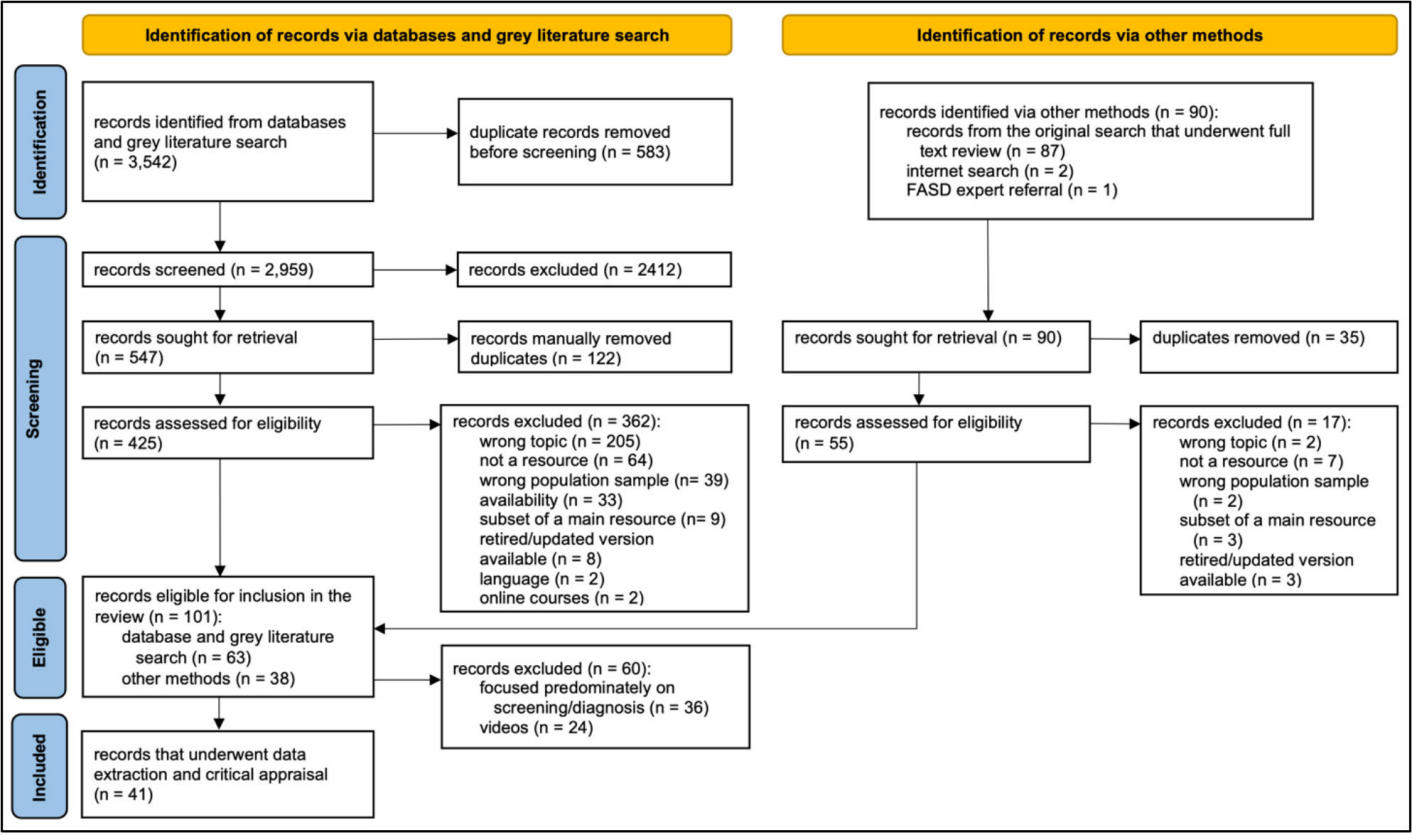
PRISMA 2020 flow diagram for resource screening and selection. FASD, fetal alcohol spectrum disorder; PRISMA, Preferred Reporting Items for Systematic Reviews and Meta-Analyses.

A total of 3542 records were identified via database and grey literature searches, and 583 duplicates were removed before screening (figure 1).^63^ The remaining records (n=2959) were screened and assessed for eligibility. 90 records were identified via other methods (including reference list screening), from which 35 duplicates were removed, and 17 records were excluded. A total of 101 records met the review’s inclusion criteria, including 63 records from the database and grey literature searches and 38 records identified by handsearching reference lists and FASD expert referral.

#### Overview of resources

The resources (n=101) eligible for inclusion in the review included guidelines (n=18, 17.8%); guides (n=30, 29.7%); factsheets (n=12, 11.9%); screening tools (n=11, 10.9%); diagnosis tools (n=6, 5.9%) and videos (n=24, 23.8%). All resources (except the videos) were categorised based on their focus on identification/screening, diagnosis, referral/management and policy/broad topics, with many assigned to more than one topic. Because systematic reviews of FASD screening tools^45 55^ and diagnostic guidelines exist^56^ or are underway,^57^ resources were later excluded if they focused predominately on screening or diagnosis (n=36; 35.6%) (see online supplemental table S5) or were videos (n=24, 23.8%) (see online supplemental table S6). Consequently, this review included guidelines, guides and factsheets covering primarily referral/management or policy/broad topics.

#### Characteristics of resources included in the review

A total of n=41 resources were included in the review: guidelines (n=9, 22.0%)^64^,^72^ guides (n=22, 53.7%)^73^,^94^ and factsheets (n=10, 24.4%)^95^,^104^ (see online supplemental table S7). Resources were published or updated between 1980 and 2022, with over half published/updated in the past ten years (n=24, 58.5%, table 2). Most resources were developed in the USA (n=15, 36.6%), Australia (n=12, 29.3%) or Canada (n=7, 17.1%). 17 (41.5%) resources were journal articles, and over half focused on at least two topics (n=27, 65.9%). Nearly all resources focused on referral or management (n=40, 97.6%), of which some were language guides (n=5, 12.5%). More than half of the resources focused on diagnosis (n=23, 56.1%) and assessment (identification/screening) (n=22, 53.7%) and about one-fifth focused on policy/broad topics (n=9, 22%) and prevention (n=9, 22%). Resources focused on one or more levels of health service, mostly face-to-face contact with patients (n=40, 97.6%), administration (n=12, 29.3%) and policy (n=8, 19.5%). Nearly half of the resources were based on literature/clinical research (n=18, 43.9%) or expert judgement and literature/clinical research (n=17, 41.5%), but five (12.2%) resources did not report an evidence base. Only five resources stated that they had obtained consumers’ (people with FASD) feedback on resources, including two guidelines,^65 66^ two guides^79 91^ and one factsheet.^104^

**Table 2:** Characteristics of resources by type

	Total (n=41)	Guidelines (n=9)	Guides (n=22)	Factsheets (n=10)
	n (%)	n (%)	n (%)	n (%)
Date				
Published/updated in 2013 or after	24 (58.5)	6 (66.7)	13 (59.1)	5 (50)
Published/updated in 2012 or earlier	13 (31.7)	3 (33.3)	8 (36.4)	2 (20)
No date provided	4 (9.8)	0 (0)	1 (4.5)	3 (30)
Format				
Report/other	24 (58.5)	6 (66.7)	12 (54.5)	6 (60)
Journal article	17 (41.5)	3 (33.3)	10 (45.5)	4 (40)
Country				
USA	15 (36.6)	3 (33.3)	9 (40.9)	3 (30)
Australia	12 (29.3)	2 (22.2)	4 (18.2)	6 (60)
Canada	7 (17.1)	0 (0)	6 (27.3)	1 (10)
UK	5 (12.2)	2 (22.2)	3 (13.6)	0 (0)
Scotland	1 (2.4)	1 (11.1)	0 (0)	0 (0)
South Africa	1 (2.4)	1 (11.1)	0 (0)	0 (0)
Topic*				
Referral/management	40 (97.6)	8 (88.9)	22 (100)	10 (100)
Diagnosis	23 (56.1)	8 (88.9)	13 (59.1)	2 (20)
Assessment	22 (53.7)	9 (100)	11 (50)	2 (20)
Broad/policy	9 (22)	6 (66.7)	3 (13.6)	0 (0)
Health service level*				
Face to face with patients	40 (97.6)	8 (88.9)	22 (100)	10 (100)
Administration	12 (29.3)	4 (44.4)	7 (31.8)	1 (10)
Policy	8 (19.5)	4 (44.4)	4 (18.2)	0 (0)
Evidence base				
Literature/clinical research	18 (43.9)	4 (44.4)	10 (45.5)	4 (40)
Expert judgement and literature/clinical research	17 (41.5)	5 (55.6)	9 (40.9)	3 (30)
None reported	5 (12.2)	0 (0)	2 (9.1)	3 (30)
Expert judgement	1 (2.4)	0 (0)	1 (4.5)	0 (0)
For use with First Nations Australians				
No	38 (92.7)	7 (77.8)	21 (95.5)	10 (100)
Yes	3 (7.3)	2 (22.2)	1 (4.5)	0 (0)
Designed for health professionals in First Nations Australian communities				
No	38 (92.7)	7 (77.8)	21 (95.5)	10 (100)
Yes	3 (7.3)	2 (22.2)	1 (4.5)	0 (0)
Designed with input from First Nations Australians				
No	38 (92.7)	7 (77.8)	21 (95.5)	10 (100)
Yes	3 (7.3)	2 (22.2)	1 (4.5)	0 (0)

* Some resources covered more than one characteristic.

Most resources were designed for a broad category of ‘health professionals’, including allied health professionals (social workers, speech and language therapists, occupational therapists and psychologists), behavioural health professionals (particularly substance abuse and mental health treatment professionals), child development specialists, clinical geneticists, counsellors, general practitioners (GPs), multidisciplinary teams, neonatologists, nurses, paediatric neuropsychologists, paediatricians, physiotherapists, physician assistants, psychiatrists and researchers. Some included information for policy-makers, programme administrators, social service providers and members of the judicial system.

The resources included outcome measures or recommendations that covered various themes related to FASD referral/management or policy/broad topics. These included behaviour, social, physical and neurological characteristics of FASD; case management; comorbidities; communication, sleep, nutrition, mental health, hearing, and dental assessment and management; education for health professionals, families and other services providers; health service enhancement; management across the lifespan; management evaluation; management plans; medical, non-medical, psychological and educational interventions; models of care; policy and advocacy considerations; recommended language around FASD; referral pathways; risk and protective factors of those living with FASD and support information and services.

#### Quality appraisal: guidelines

All guidelines had an overall score of 76%–100% for scope and purpose (domain 1) (n=9, 100%) and about half scored between 76% and 100% for stakeholder involvement (domain 2) (n=5, 55.6%), rigour of development (domain 3) (n=4, 44.4%) and clarity of presentation (domain 4) (n=5, 55.6%). Although one-third of guidelines scored either 26%–50% (n=3, 33.3%) or 51%–75% (n=3, 33.3%) for applicability—Australian context (domain 5 a), most scored 100% for editorial independence (domain 6) (n=7, 77.8%). Over half of the guidelines scored 76%–100% for overall quality (n=5, 55.6%). One author (TS) deemed that most guidelines could be recommended for use in the Australian context with modifications (n=7, 77.8%), and two published before 2010 were deemed outdated (n=2, 22.2%). The overall domain and quality assessment scores for the guidelines using the AGREE II instrument are provided (table 3). For the scores provided for each of the AGREE II instrument’s 23 items, see online supplemental table S8.

**Table 3:** Overall quality scores and domain scores of guidelines using the AGREE II instrument

Resource reference	Domain 1	Domain 2	Domain 3	Domain 4	Domain 5a	Domain 5b*	Domain 6	Overall quality
Adebiyi *et al,* 2019^48^	94	50	38	61	17	0	100	51
Bertrand *et al,* 2004^65^	94	94	81	67	63	0	0	72
Bertrand *et al,* 2005^65^	83	83	23	61	42	0	100	54
NACCHO and RACP 2018^67^	94	89	83	78	46	42	100	80
Lim *et al,* 2021^68^	100	61	83	94	71	58	100	83
National FASD 2019^69^	78	83	81	89	54	0	100	79
SIGN 2019^70^	89	94	77	78	79	0	100	83
SAMHSA 2014^71^	94	61	75	78	88	0	58	77
Young *et al* 2016^72^	94	61	48	72	54	0	100	64

Domain 1: Scscope and purpose; Ddomain 2: Sstakeholder involvement; Ddomain 3: Rrigour of development; Ddomain 4: Cclarity of presentation; Ddomain 5aa: Aapplicability—Australian context; Ddomain 5b: Applicability— First Nations Australians; Ddomain 6: Eeditorial independence.

* Scores on domain 5b were not included in overall assessment scores.

AGREE IIAppraisal of Guidelines for REsearch and Evaluation IINACCHONational Aboriginal Community Controlled Health OrganisationRACPRoyal Australasian College of PhysiciansSAMHSASubstance Abuse and Mental Health Services AdministrationSIGNScottish Intercollegiate Guidelines Network

The quality appraisal scores of guides (n=22) and factsheets (n=10) are listed in table 4 (and online supplemental table S9). Most guides (n=17, 77.3%) and factsheets (n=7, 70.0%) scored ‘good’ on evidence base (item 1), and all guides (n=22, 100%) and factsheets (n=10, 100%) scored ‘excellent’ on generalisability (item 2). All guides (n=22, 100%) and factsheets (n=10, 100%) scored either ‘excellent’ or ‘good’ for applicability to patient populations—Australia (item 4a) and credibility (item 8). However, around one-third of guides (n=8, 36.3%) and one-fifth (n=2, 20%) of factsheets scored ‘satisfactory’ or ‘poor’ on timing (currency) (item 6). Most guides (n=21, 95.4%) and factsheets (n=7, 70%) scored ‘excellent’ for ease of use, while most guides (n=15, 68.2%) and half the factsheets (n=5, 50%) scored ‘excellent’ for credibility.

**Table 4:** Item scores for guides and factsheets based on the modified NHMRC and iCAHE appraisal tool

Resource reference	Item 1	Item 2	Item 3	Item 4a	Item 4b*	Item 5	Item 6	Item 7	Item 8
American Academy of Pediatrics 2022^73^	C	C	A	B	B	A	A	A	A
Bartlett and Davis^74^	C	B	A	B	B	B	D	A	B
British Medical Association 2007^75^	B	B	A	B	B	A	A	A	A
Brown *et al* 2018^76^	B	B	A	B	B	A	A	A	B
Chudley and Longstaffe^77^	B	B	A	B	B	A	C	A	A
Dudley *et al* 2016^78^	D	B	A	A	A	A	B	A	B
FASD Hub Australia 2019^79^	B	B	A	A	B	A	A	A	A
US DHHS, CDC 2015^80^	B	B	A	B	B	A	B	A	A
Fleming 1999^81^	B	B	A	B	B	A	D	A	A
FARE n.d.^82^	B	C	A	A	A	A	D	A	A
Gray and Mukherjee^83^	B	C	A	B	B	A	C	D	A
Hagan *et al* 2016^84^	B	C	A	B	B	A	B	A	A
Hanlon-Dearman *et al* 2015^85^	B	B	A	A	B	A	B	A	A
Burd 2013^86^	B	C	A	B	B	A	B	A	A
Canada Northwest FASD Partnership 2016^87^	D	B	A	B	B	A	A	A	B
Martyniuk and Melrose^88^	C	C	A	B	B	A	A	A	A
Ozsarfati and Koren^89^	B	B	A	B	B	A	B	A	B
Peadon and Elliott^90^	B	B	A	A	A	A	B	A	A
Seashell and NOFASD UK 2020^91^	B	B	A	B	B	A	A	A	A
Sokol and Clarren^92^	B	D	A	B	B	A	D	A	B
Todorow *et al* 2012^93^	B	C	A	B	B	A	C	A	A
Wilton and Plane^94^	B	C	A	B	B	A	D	A	B
Cannon *et al* 2020^95^	B	C	A	A	A	A	A	A	B
Cannon *et al* 2020^96^	B	C	A	A	A	A	A	A	B
Kippin *et al* 2020^97^	B	C	A	A	A	A	A	A	B
Green *et al* 2001^98^	B	C	A	B	B	B	D	D	A
Huggins *et al* 2008^99^	B	C	A	B	B	A	C	D	A
Nash and Davies^100^	B	C	A	B	B	A	B	C	B
NOFASD Australia^101^	D	C	A	A	B	A	A	A	B
NOFASD Australia^102^	D	C	A	A	B	A	A	A	A
NOFASD Australia^103^	D	C	A	A	B	A	A	A	A
Rutman 2016^104^	B	C	A	B	B	A	B	A	A

Item 1: Eevidence base; Iitem 2: Cclinical impact; Iitem 3: generalisability to health professionals working with FASD; Iitem 4a: Aapplicability to patient populations—Australia; Iitem 4b: Aapplicability to patient populations—First Nations Australians; Iitem 5: Aavailability; Iitem 6: timing and updates/review (currency); Iitem 7: Eease of use; Iitem 8: Ccredibility.

Scores: A=excellent; B=good; C=satisfactory; D=poor.

* Scores for item 4b were not included in overall scores.

CDCCenters for Disease Control and PreventionFAREFoundation for Alcohol Research and EducationFASDfetal alcohol spectrum disordern.dno dateNHMRCNational Health and Medical Research CouncilUSDHHSUS Department of Health and Human Services

#### Resources for First Nations Australian communities

Only three resources (7.3%), including two guidelines and one guide, were designed specifically: (a) for use with First Nations Australians, (b) for health professionals in First Nations Australian communities or (c) with input from First Nations Australians.^67 68 78^ On quality appraisal, the two guidelines scored 58.3% and 41.7% for applicability—First Nations Australians (domain 5b).^67 68^ The guide scored ‘excellent’ for applicability to patient populations—First Nations Australians (item 4b).^78^ Although not explicitly designed for use with or input from First Nations Australian populations, all other guides (n=21) and all factsheets (n=10) scored ‘excellent’ or ‘good’ for applicability to patient populations—First Nations Australians (item 4b).

## Discussion

This scoping review identified, analysed and critically appraised publicly accessible resources for health professionals to refer or support people with FASD. It also evaluated the appropriateness of the resources for health professionals working in First Nations Australian communities. A total of 101 resources were identified, of which 41 underwent data extraction and critical appraisal. All are current and suitable to the specific service settings and roles in which health professionals work with people with FASD and their families. Most were published or updated in 2013 or later, covered various aspects of the health system (face to face with patients, administration, policy) and were based on evidence from research literature or expert judgement and evidence from the research literature. The characteristics of resources included in this review may aid health professionals in identifying the type and content of resources most relevant to their specific settings and patient profiles. Further, the resources identified in this review are more applicable to health professionals in their roles in healthcare settings than the resources identified in previous reviews that focused on FASD resources for use in the general community^44^,^48^ or by education professionals.^50^

The current review focused on resources for referring or supporting people with FASD and policy/broad topics. Given this focus, health professionals could use these resources alongside resources identified in previous reviews that focused on FASD screening tools^45 55^ and diagnostic guidelines^56^ or in a review currently underway on FASD diagnostic guidelines.^57^ Our review and others address critical gaps in the literature concerning a lack of educational FASD resources and standardised tools purposefully designed for health professionals.^1^ PAE can affect all aspects of the body and brain,^105^ making FASD a heterogeneous disorder that requires contact with a range of health professionals. As such, most resources were aimed toward broadly defined ‘health professionals’. Although these resources were widely accessible, the content typically provided only brief overviews of broad topics, such as characteristics and symptoms of FASD, models of care, strategies for supporting people with FASD or language/terminology. A few resources focused on allied health professionals (occupational therapists, speech and language therapists, social workers), mental health professionals (psychiatrists, psychologists, behavioural specialists) and those engaged at the policy-making, health system and programme levels. These targeted resources tended to provide more in-depth, discipline-specific information. For example, there was a psychiatrist’s guide for managing the psychiatric and neurodevelopmental disorders of FASD. The limited symptom-specific or clinician-specific resources likely reflect the paucity of research on evidence-based management strategies for FASD, particularly in comparison to other neurodevelopmental disorders, like autism spectrum disorder. Future research is needed to address this evidence gap and improve understanding of FASD-specific treatments and management strategies for common functional impairments in FASD and development of evidence-based, clinician-specific resources by experts in the field would also be valuable.

Only five of the resources reportedly obtained input from people living with FASD, including two guidelines,^65 66^ two guides^79 91^ and one factsheet.^104^ This is a crucial weakness of the current resources that should be addressed by increased efforts to capture and intergrate the perspectives of those with lived experience of FASD into resources for health professionals. This apporach may help ensure that the development and deployment of resources are better aligned with contexts, needs and preferences of people with FASD and their caregivers and families.^49^ People with lived experience of FASD should also be invited to provide input, through consultation or codesign, into policies, programmes and services focused on prevention, screening, diagnosis and management/supports.

This review identified high-quality resources to assist health professionals in engaging with patients and families with FASD. Nearly all resources included information on working face to face with patients, most guidelines were recommended for use in the Australian context with modifications, and all the guides and factsheets scored ‘good’ or ‘excellent’ in terms of generalisability and credibility. However, some resources were outdated and scored low on specific criteria, such as rigour of development and clarity of presentation. The literature shows that health professionals have an essential role in FASD prevention^34^ and in delivering integrated care and support for individuals with FASD and their families.^35^ Consequently, the resources, despite their limitations, may help address an important gap in health professionals’ knowledge, skills and confidence to engage with patients and families on FASD-related topics, incuding their hesitancy to provide screening, diagnosis, support and prevention services.^3638^,^40 106^ Although research has shown that the distribution of FASD prevention resources was well received by paediatricians in Australia, it had limited impact on their knowledge and practices—highlighting the limitation of educational resources alone to change practice.^37^ Moreover, some resources for working face to face with patients included language guides on FASD that may be helpful for health professionals.^79 87 88 91 92^ Using appropriate language about FASD is important for creating a respectful, non-judgemental and non-stigmatising environment to discuss PAE^1^ and provide integrated care and support services to people with FASD and their families.^79^ Most of the language guides on FASD were recently updated, but one published over ten years ago includes out-of-date language.^92^ As such, health professionals should only use terminology in current best-practice guides.

Although the scoping review’s findings have implications for health professionals globally, they are particularly relevant to enhancing their role in improving FASD-related outcomes in Australia. Nearly one-third of the resources were published in Australia (n=12, 29.3%), including some guidelines (n=2, 22.2%), and guides (n=4, 18.2%), and most of the factsheets (n=6, 60.0%). Moreover, both guidelines published in Australia were developed by reputable organisations^67 68^ and all the guides and factsheets published in Australia scored ‘excellent’ for applicability to Australian patient populations. The resources published in other high-income or middle-income countries, with a similar socioeconomic environment to Australia, may be relevant in this context; however, they may not be directly relevant to countries with different health systems, such as the USA or low-income countriess. These findings align with the Australia National FASD Strategic Action Plan 2018–2028 that aims to (1) reduce the prevalence of FASD, (2) reduce the associated impacts of FASD and (3) improve the quality of life for people with FASD, including through increasing access to appropriate diagnostic and support services to improve care and outcomes for people with FASD and provide education and training for health and community service providers to ensure they have the knowledge and confidence to diagnose and support people with FASD.^33^

Although the resources align with the Australian context at the national level, they are less suitable for First Nations Australians, a priority population in the Australia National FASD Strategic Action Plan 2018–2028.^33^ Given the high prevalence of PAE and FASD in some remote First Nations Australian communities,^17^,^19^ and the significant adverse impacts of FASD on child neurodevelopment,^20^,^24^ education attainment^25^ and hospital admission rates,^26^ support for health professionals in these settings is an imperative. However, only 3 (7.3%) of the 41 resources identified (2 guidelines and 1 guide) were designed for use with First Nations Australians, for health professionals in First Nations Australian communities, or with input from First Nations Australians.^67 68 78^ On quality appraisal, these two guidelines received overall scores of 58.3% and 41.7% for applicability to First Nations Australian patient populations and the guide scored ‘excellent’ for this domain. Despite their limitations, these and other culturally appropriate resources for FASD prevention among First Nations Australians^107^ would be useful for health professionals in these settings. However, this study highlights a gap in resources for health professionals supporting First Nations communities.

High-quality resources may assist health professionals to prevent or reduce the potential harms from PAE through improved FASD education, screening/diagnosis, referral and support services for key populations, with early intervention reducing the risk of negative impacts associated with FASD later in life.^29^,^32^ Additionally, to improve outcomes for First Nations Australians with FASD, it is crucial to acknowledge and address the significant, ongoing impacts of colonisation and intergenerational trauma on their health and well-being,^13^,^16^ and the barriers to health service access that hinder efforts to address these increased needs.^27 28^ Further, cross-cultural barriers heighten challenges of health service provision for First Nations Australians, with some health professionals and support workers reporting a lack of understanding of culturally appropriate ways to engage about FASD.^41^ Thus, the Australia National FASD Strategic Action Plan 2018–2028 calls for culturally appropriate FASD prevention and support for First Nations Australian communities, drawing on successful examples across Australia.^33^ Relatedly, the Australian FASD Indigenous Framework argues for development of more First Nations-grounded, strengths-based, healing-informed approaches—that are based on holistic and integrated support—into existing health services for FASD with First Nations Australians, including how health professionals engage in communities.^42^

## Limitations

This scoping review has several limitations that should be considered alongside the findings. First, the study only included resources published in English. This is an important limitation because non-English resources would be crucial for health professionals working in various settings, including with specific minority or marginalised groups. Second, videos were excluded because the data extraction and critical appraisal tools used were developed for written resources and were unsuitable. This does not discount the potential value of video resources for health professionals supporting people with FASD and their families. Third, the two coauthors who extracted data and critically appraise all resources did not compare all their appraisals; however, inter-rater reliability and agreement were high for the random sample of resources assessed by two coauthors. Fourth, authors of resources were not contacted to clarify their development process, including the involvement of stakeholders including people with lived experience of FASD. Some resources may have been developed using this consultation process, even though this information was not provided. Fifth, although the review included input from FASD experts and several coauthors who had indirect lived experience of FASD (EC, ST, EJE, LJR and ALCM), including in remote First Nations Australian communities, input from those with lived experience of FASD was limited. Future studies would benefit from including their perspectives. Sixth, this review excluded resources that predominately focused on FASD screening or diagnostic guidelines because systematic reviews on these resources exist^45 55 56^ or are underway.^57^ This narrow focus is a potential limitation as some health professionals may want easy access to different resources in one location and based on their specific needs and patient populations. We would direct health professionals to access published and ongoing reviews for information on screening and diagnostic tools and this review for resources on primarily referral/management or policy/broad topics. Despite these limitations, this is the first review of its kind, and it provides valuable information to inform health policy and education strategies for health professionals.

## Conclusions

People with FASD have an increased risk of poor educational, social and health outcomes in early and later life. Health professionals are suited to deliver FASD prevention, screening, and diagnosis as well as to provide support to people with FASD and their families. However, they often lack the knowledge, confidence and resources to deliver these services, particularly in cross-cultural settings. This scoping review identified high-quality guidelines, guides and factsheets to support health professionals in providing referrals and support for FASD, including various guides on the appropriate and respectful use of language on FASD. Resources covering FASD screening and diagnosis are published elsewhere, but the resources identified in this study will assist health professionals to provide integrated care for people with FASD and their families. Since the scoping review identified only three FASD resources developed for use in and with input from First Nations Australians, efforts are needed to better assist health professionals to effectively support these communities. People living with FASD should lead the codesign of new resources to ensure their perspectives and preferences are captured and integrated.

## supplementary material

10.1136/bmjopen-2024-086999 online supplemental file 1

## Data Availability

All data relevant to the study are included in the article or uploaded as online supplemental information.
