# Supplementary material for: Fetal alcohol spectrum disorder resources for health professionals: a scoping review
Source: BMJ Open. 2024 Jul 12;14(7):e086999. doi: 10.1136/bmjopen-2024-086999 (PMC11253770; doi:10.1136/bmjopen-2024-086999)
Supplement: Supplementary file 2 [file bmjopen-2024-086999.reviewer_comments.pdf]

PEER REVIEW HISTORY

BMJ Open publishes all reviews undertaken for accepted manuscripts. Reviewers are asked to complete a checklist review form (**http://bmjopen.bmj.com/site/about/resources/checklist.pdf**) and are provided with free text boxes to elaborate on their assessment. These free text comments are reproduced below.

ARTICLE DETAILS

**TITLE (PROVISIONAL)**	Fetal Alcohol Spectrum Disorder Resources for Health Professionals: A Scoping Review
**AUTHORS**	Stubbs, Thomas; Cannon, Lisa; Carter, Emily; Naanai, Habiba; Okurame, Josephine; Martiniuk, Alexandra; Davies, Jadnah; Thomas, Sue; Bedford, Mudge; Elliott, EJ; Rice, Lauren

VERSION 1 – REVIEW

**REVIEWER**	Millians, Molly<br>Emory University, Psychiatry and Behavioral Sciences
**REVIEW RETURNED**	15-Apr-2024

**GENERAL COMMENTS**	The article “Fetal Alcohol Spectrum Disorder Resources for Health Professionals: A Scoping Review” provides a well written appraisal of the available materials for health professionals working with individuals with Fetal Alcohol Spectrum Disorders (FASD).<br>It appears that focus of the current article differed from the2022 article that outlined the protocol used for this review (see Okurame et al., 2022). The current article added an aim that narrowed the focus of the review. The article’s abstract, discussion, and conclusion predominately discussed materials and issues related to resources about FASD for health professionals working with Aboriginal and Torres Strait Islander Peoples in Australia . The article does attempt to embed it within a larger review of resources related to FASD for health providers, many of which are developed in the United States. From the viewpoint of this reviewer, the title of the current article fails to capture the focus of the article related to resources for healthcare providers working with indigenous populations in Australia. It might be beneficial to consider

	revising the article's title to better reflect its main focus.<br><br>Okurame, J. C., Cannon, L., Carter, E., Thomas, S., Elliott, E. J., & Rice, L. J. (2022). Fetal alcohol spectrum disorder resources for health professionals: a scoping review protocol. *BMJ Open*, *12*(9), e065327. https://doi.org/10.1136/bmjopen-2022-065327

**REVIEWER**	Tenenbaum , Ariel<br>Hadassah Medical Center, Pediatrics
**REVIEW RETURNED**	04-May-2024

**GENERAL COMMENTS**	I thank the editor for the opportunity to review this interesting article on FASD resources for health professionals. I congratulate the authors for the comprehensive and thorough work.<br>This study is focused on a specific aspect of FASD which is the existing resources in English that are available for professionals.<br>I found all segments of the manuscript well written and informative, and I do not have any significant editing (or other) remarks on the text. The authors presented the aims of the study and I think that they did very well in answering all the questions, and the conclusions are very clear.<br><br>My questions and suggestions to the authors regarding the manuscript are:<br><br>1. The subject of the study is somewhat narrow and specific. I suggest that the authors elaborate more on the knowledge gap and the importance and possible impact of the questions and answers that the study presents.<br>2. The authors included only comprehensive resources. For example, they excluded resources that include only screening tools or diagnostic guidelines. I suggest that the authors explain why they chose to do so. Physicians may look for specific items or data on FASD according to the information that they seek. For example, they may want a good screening tool and do not need a comprehensive tool that might be too long or complex. In some cases, the specific item on a specific aspect of FASD can be more useful.<br>3. Quantity and quality: the authors count the resources and score them. I have a basic question on this topic – If there is one resource that is excellent and easily accessible – can't it be enough? I'm not sure that the number of items is really telling us that much.<br>4. The authors state that there is a need for more appropriate resources for supporting Indigenous Australian communities. They also state in methods and limitations that they included only resources in English. In this aspect, I have a comment regarding the possible generalization of the study (and it is not about the Indigenous Australian communities or any specific group): If all non-English resources are excluded, it is difficult to assess the availability of good resources that may exist and were intentionally written in specific languages for minority and/or marginalized groups. I suggest that the authors address this limitation of the study in more details.

VERSION 1 – AUTHOR RESPONSE

Response to reviewer comments

**Reviewer 1**	**Response**
It appears that focus of the current article differed from the2022 article that outlined the protocol used for this review (see Okurame et al., 2022). The current article added an aim that narrowed the focus of the review. The article's abstract, discussion, and conclusion predominately discussed materials and issues related to resources about FASD for health professionals working with Aboriginal and Torres Strait Islander Peoples in Australia. The article does attempt to embed it within a larger review of resources related to FASD for health providers, many of which are developed in the United States. From the viewpoint of this reviewer, the title of the current article fails to capture the focus of the article related to resources for healthcare providers working with indigenous populations in Australia. It might be beneficial to consider revising the article's title to better reflect its main focus.	We acknowledge and address the reviewer's feedback by refocusing the manuscript on the original objectives outlined in the protocol for this review (see Okurame et al., 2022).<br><br>To this end, we have shifted the focus of the introduction/literature review to health professionals working with people living with FASD in all healthcare setting and minimised the focus on Indigenous Australian communities. Further, in the results section we have added additional information on the characteristics of the resources to demonstrate their international applicability. This includes information about the health professionals who are intended recipients of the resources; and the overall topics covered by the outcome measures/recommendations in the resources.<br><br>Further, we have highlighted the current gaps identified by the review as they related to health professionals working with people living with FASD in all healthcare settings, including our recommendations. The sections on First Nations and FASD have been reduced in the discussion and conclusion.
**Reviewer 2**	**Response**
1. The subject of the study is somewhat narrow and specific. I suggest that the authors elaborate more on the knowledge gap and the importance and possible impact of the questions and answers that the study presents.	We acknowledge and address the reviewer's comment by elaborating on the knowledge gap addressed by this review and its importance and potential impact. To this end, we have added several sentences to the middle of the last paragraph of the "Introduction" section of the manuscript. This section now summarises and highlights the current research on the important role of health professionals in FASD prevention and support for families, but their limited capacity and confidence to do so in some cases. It also highlights the high prevalence and burden of FASD on minority and marginalised populations as an important public health

	priority. Further, it highlights the potential public health gains that might be achieved by better supporting health professionals to engage in FASD prevention and support.
2. The authors included only comprehensive resources. For example, they excluded resources that include only screening tools or diagnostic guidelines. I suggest that the authors explain why they chose to do so. Physicians may look for specific items or data on FASD according to the information that they seek. For example, they may want a good screening tool and do not need a comprehensive tool that might be too long or complex. In some cases, the specific item on a specific aspect of FASD can be more useful.	We address the reviewer's comment on this manuscript's exclusion of resources that only focus screening and diagnostic guidelines.<br><br>First, we added new text in the "Stage 4-5: Screening and Selection" section of the manuscript to explain systematic reviews on screening tools and diagnostic guidelines currently exist or are underway. We also add text to explain that health professionals seeking information on these resources can obtain it at these current and future publications. This additional information also helps to justify why the coauthors excluded screening and diagnostic tools from this review.<br><br>Second, we have added new text at the bottom of the "Limitations" section of the manuscript, highlighting how excluding resources on screening and diagnostic guidelines is a limitation of this scoping review, because it means health professionals cannot access information on all types of FASD resources in the one publication. However, we also address this limitation by directing health professionals to the current and future publications covering these resources.
3. Quantity and quality: the authors count the resources and score them. I have a basic question on this topic – If there is one resource that is excellent and easily accessible – can't it be enough? I'm not sure that the number of items is really telling us that much.	We acknowledge and address the reviewer's comment regarding the limited value of summarising the quantity of resources, which is covered in the "2. Overview of resources" section of the manuscript.<br><br>However, the coauthorsfeel there is value in the describing the published date, country of development, specific focus, evidence base, inclusion of consumers' feedback of the FASD resources—which is outlined in the "3. Characteristics of resources included in the review" section of the manuscript—as it provides health professionals with information about the resources that are most relevant to

	their specific setting. This strength has been noted through new text added to the end of the first paragraph of the discussion.<br><br>The coauthors also feel there is value in describing the quality of resources—which is covered in the “4. Quality appraisal: guidelines” and “5. Quality appraisal: guides & factsheets”—as the quality of resources varied a lot and this information would be useful to health professionals to identify and use the highest quality resources. We have highlighted this strength by adding new text to the bottom of the third paragraph in the discussion.<br><br>Further, the “6. Resources for Indigenous Australian communities” section of the manuscript is particularly important for health professionals working alongside Australian First Nations communities, as the previous literature has shown that health professionals often lack confidence and cultural understanding to engage with communities in these settings. This important strength has been elaborated on with new text at the bottom of the eighth paragraph of the discussion.
4. The authors state that there is a need for more appropriate resources for supporting Indigenous Australian communities. They also state in methods and limitations that they included only resources in English. In this aspect, I have a comment regarding the possible generalization of the study (and it is not about the Indigenous Australian communities or any specific group): If all non-English resources are excluded, it is difficult to assess the availability of good resources that may exist and were intentionally written in specific languages for minority and/or marginalized groups. I suggest that the authors address this limitation of the study in more details.	We acknowledge and address the reviewer’s feedback in two ways.<br><br>First, we have added text in the “Stage 2: Inclusion Criteria” section of the manuscript to highlight that published in English was an inclusion criterion of this study and explained that this meant non-English resources, including those for specific minority or marginalised groups, would not captured in this scoping review. The text also notes that this limitation is addressed below.<br><br>Second, we have added text in the “Limitations” section of the manuscript to elaborate that the published in English inclusion criteria was a major limitation of this scoping review. The text also highlights how this scoping review doesn’t include resources for health professionals in

	non-English settings, including those working with some minority or marginalised groups. Further, the text calls for further studies that specifically focus on non-English FASD resources for health professionals working in non-English speaking settings.

VERSION 2 – REVIEW

**REVIEWER**	Tenenbaum , Ariel<br>Hadassah Medical Center, Pediatrics
**REVIEW RETURNED**	05-Jun-2024

**GENERAL COMMENTS**	I have no further comments. Thank you.
